# A Plaque Disruption Index Identifies Patients with Non-STE-Type 1 Myocardial Infarction within 24 Hours of Troponin Positivity

**DOI:** 10.1371/journal.pone.0164315

**Published:** 2016-10-06

**Authors:** Maha A. Al-Mohaissen, Ronald G. Carere, G. B. John Mancini, Karin H. Humphries, Beth A. Whalen, Terry Lee, Frank X. Scheuermeyer, Andrew P. Ignaszewski

**Affiliations:** 1 Department of Clinical Sciences, Princess Nourah bint Abdulrahman University, Riyadh, Saudi Arabia; 2 Department of Medicine, Division of Cardiology, St. Paul's Hospital and the University of British Columbia, Vancouver, BC, Canada; 3 Department of Medicine, University of British Columbia, Vancouver, BC, Canada; 4 Centre for Heart Lung Innovation, St. Paul's Hospital and University of British Columbia, Vancouver, BC, Canada; 5 Centre for Health Evaluation and Outcome Sciences, St. Paul’s Hospital and University of British Columbia, Vancouver, BC, Canada; 6 Department of Emergency Medicine, St. Paul's Hospital and the University of British Columbia, Vancouver, BC, Canada; Medstar Washington Hospital Center, UNITED STATES

## Abstract

**Background:**

Markers of plaque destabilization and disruption may have a role in identifying non-STE- type 1 Myocardial Infarction in patients presenting with troponin elevation. We hypothesized that a plaque disruption index (PDI) derived from multiple biomarkers and measured within 24 hours from the first detectable troponin in patients with acute non-STE- type 1 MI (NSTEMI-A) will confirm the diagnosis and identify these patients with higher specificity when compared to individual markers and coronary angiography.

**Methods:**

We examined 4 biomarkers of plaque destabilization and disruption: myeloperoxidase (MPO), high-sensitivity interleukin-6, myeloid-related protein 8/14 (MRP8/14) and pregnancy-associated plasma protein-A (PAPP-A) in 83 consecutive patients in 4 groups: stable non-obstructive coronary artery disease (CAD), stable obstructive CAD, NSTEMI-A (enrolled within 24 hours of troponin positivity), and NSTEMI-L (Late presentation NSTEMI, enrolled beyond the 24 hour limit). The PDI was calculated and the patients’ coronary angiograms were reviewed for evidence of plaque disruption. The diagnostic performance of the PDI and angiography were compared.

**Results:**

Compared to other biomarkers, MPO had the highest specificity (83%) for NSTEMI-A diagnosis (P<0.05). The PDI computed from PAPP-A, MRP8/14 and MPO was higher in NSTEMI-A patients compared to the other three groups (p<0.001) and had the highest diagnostic specificity (87%) with 79% sensitivity and 86% accuracy, which were higher compared to those obtained with MPO, but did not reach statistical significance (P>0.05 for all comparisons). The PDI had higher specificity and accuracy for NSTEMI-A diagnosis compared to coronary angiography (P<0.05).

**Conclusions:**

A PDI measured within 24 hour of troponin positivity has potential to identify subjects with acute Non-ST-elevation type 1 MI. Additional evidence using other marker combinations and investigation in a sufficiently large non-selected cohort is warranted to establish the diagnostic accuracy of the PDI and its potential role in differentiating type 1 and type 2 MI in patients presenting with troponin elevation of uncertain etiology.

## Introduction

The increasing sensitivity of cardiac troponins (cTn) came at the cost of reduced clinical specificity for the diagnosis of spontaneous myocardial infarction (type 1 MI) [1], leading to diagnostic confusion and an augmented work burden to identify “clinically false positive” events. Proposing higher cTn cutoffs [2; 3], calculating the delta troponin criterion, [4] and incorporating clinical predictors [5] and other cardiac tests in the interpretation of cTn results [2] have been suggested, but remain suboptimal and impractical [6; 7].

Differentiating type 1 MI from non-ACS related cTn elevations [8] is an increasingly encountered diagnostic dilemma [9]. Markers of plaque destabilization and disruption, being of coronary origin, [10] may be of value in that regard by confirming acute NSTE-type 1 MI (NSTEMI-A) in patients with cTn elevation. However, their diagnostic potential in distinguishing Type 1 MI has not been evaluated, and therefore there is ambiguity about the optimal sampling time in ACS, and which biomarker to use. Additionally, these markers are characterized by their upstream rise [11; 12], short half-lives [12], variable release patterns [13] and reduced specificity for cardiac tissue [14] which may affect their diagnostic value. Thus, although many of these biomarkers hold promise, more evaluation is warranted [11].

When compared to cTn, a marker of myocardial necrosis, markers of plaque disruption show inferior diagnostic performance but their use as adjuncts to cTns to confirm a Type 1 MI has not been evaluated. We hypothesized that a plaque disruption index (PDI) derived from a combination of markers of plaque destabilization and disruption, measured within 24 hour of cTn positivity, will yield higher specificity and negative predictive value (NPV) in comparison to individual biomarkers and will serve as a useful adjunct to cTns in confirming the diagnosis of NSTEMI-A. We also compared the diagnostic accuracy of the PDI to that of coronary angiography, a commonly used test in cases of troponin elevation of unclear etiology, in confirming type 1 MI.

We studied 4 markers of plaque destabilization and disruption: myeloperoxidase (MPO)[11; 15], high-sensitivity interlukin-6 (hsIL6) [16; 17], myeloid-related protein 8/14 (MRP8/14) [18] and pregnancy-associated plasma protein-A (PAPP-A) [11; 19]. These markers have been (1) detected at the site of disrupted plaques; (2) their systemic concentrations are elevated in patients with ACS; and (3) cutoff values distinguishing ACS from stable CAD have been reported [18–21] with the exception of IL-6. Significant elevations of IL-6 have been reported, however, in ACS [22] and the marker has a relatively long half-life [23]. The diagnostic value of all these biomarkers in delayed ACS presentation has not been evaluated.

## Methods

### Study Population

A prospective cohort study was conducted at St Paul’s Hospital, Vancouver, British Columbia. Consecutive patients, ≥19 years of age were included in the study. The subjects were recruited from the cardiac catheterization laboratory, cardiology wards, emergency department and coronary care unit. The Providence Health Care Research Ethics Board approved this study and all patients provided written informed consent.

Four patient populations were enrolled: (1) Stable non-obstructive coronary artery disease (SNOCAD; stable patients with no symptoms suggestive of an ACS and having <50% stenosis in any major coronary artery on their coronary angiography); (2) Stable obstructive coronary artery disease (SOCAD; stable patients with no ACS symptoms and coronary stenosis of ≥50% in any major coronary artery on coronary angiography; (3) Acute non-STE-type 1 MI (NSTEMI-A) defined as presentation with typical chest pain *and* positive cTnI assay with or without ischemic ECG [8]. These patients were enrolled within a time limit specified as 24 hours from the first detectable cTnI regardless of chest pain onset and (4) Late presentation non-STE-type 1MI (NSTEMI-L, patients who were enrolled beyond 24 hours) The NSTEMI-L patients were included to study the effect of time and concomitant therapy on biomarker level.

Patients with STEMI upon presentation or having medical conditions known to cause elevations of the tested biomarkers or potentially causing type 2, 3, 4 or 5 MI were excluded. These conditions included organ transplant, hematological malignancies, connective tissue disease, inflammatory bowel disease, multiple sclerosis, recent (within 30 days) known or suspected systemic thromboembolic disease (not of coronary origin), recent surgery, pregnancy, prior percutaneous coronary intervention and or recent treatment with GPIIb/IIIa inhibitors. Patients with resuscitated cardiac arrest, hemorrhage, hypotension, severe hypertension, uncontrolled arrhythmias, decompensated heart failure, respiratory failure, myocardial contusion, hypertrophic cardiomyopathy, severe aortic stenosis, rhabdomyolysis, sepsis, cocaine use and renal failure requiring hemodialysis were also excluded.

### Data collection

After written informed consent, the patients’ baseline characteristics, (demographic data, symptoms, risk factors for cardiovascular disease and medications) and investigation results, including left ventricular ejection fraction (LVEF) and serum creatinine level were recorded.

#### Blood sampling and biomarkers measurement

Blood samples were collected from peripheral veins by phlebotomists prior to coronary angiography and arterial puncture. Serum and plasma samples were collected in serum separator and heparin tubes, respectively. The samples were then divided in aliquots and stored at -80°C until testing. Sample analysis began after 90% of the study subjects were enrolled.

We studied 4 biomarkers: MPO, MRP8/14, PAPP-A and hsIL6 in addition to cTnI. MRP8/14, PAPP-A, hsIL6 and cTnI levels were measured in serum samples and MPO was assessed in plasma samples. cTnI analysis was performed at the hospital central laboratory using the Centaur® TnI Ultra assay, a concentration>0.04 μg/L was considered positive. The analysis of the remaining biomarkers was performed at the iCapture research laboratory at SPH. We used the BÜHLMANN MRP8/14 enzyme-linked immunosorbent assay (ELISA) kit for MRP8/14 detection. PAPP-A was detected using the Diagnostic Systems Laboratories, active cPAPPA assay. HsIL-6 was measured using the Qwantikine HS human IL-6 kit and MPO was measured using the Cleveland heartlab *cardio*MPO^TM^ Enzyme Immunoassay Reagent Kit.

#### Coronary Angiograms

The patients underwent clinically indicated standard coronary angiography within 24 hours of enrolment (one NSTEMI-A patient had a coronary angiogram within 48 hours and one patient had an angiogram recently and was not referred for repeat angiography, both patients were included in the analysis). The angiograms were analyzed in the Cardiovascular Imaging Research Core Laboratory (CIRCL), Vancouver, Canada, supervised by 2 experienced cardiologists blinded to the patients' clinical data and blood test results. Diameter stenosis (DS) was measured by quantitative coronary angiography. Vessel disease was defined as the number of coronary arteries containing at least 1 lesion ≥50% DS. The COURAGE jeopardy score was calculated [24]. The angiograms were further analyzed for 6 angiographic features previously associated with culprit lesions: (1) *Thrombus formation*: acute total occlusion; and sub-totally occlusive: filling defect, haziness in the absence of calcifications or inhomogeneous opacification (2) *Intimal flap*: a radiolucent extension of the vessel wall into the arterial lumen, (3) *Ulceration*: a small crater consisting of a discrete luminal widening and luminal irregularity, (4) *Lumen irregularity*: an irregular lumen border that was not classified as ulceration (Ambrose type 2 and lesions with multiple irregularities), (5) *Impairment in wall motion* in the supplied territory, and (6) *Flow impairment* distal to the lesion [18; 25].

The following angiographic diagnoses were considered: SNOCAD, SOCAD and NSTEMI-A (Type 1 MI) were recorded based on DS and presence or absence of culprit lesion criteria. SNOCAD was defined as the presence of coronary stenosis <50% in the absence of features suggestive of a culprit lesion, SOCAD was defined as the presence of coronary stenosis ≥50% in any major coronary in the absence of features suggestive of a culprit lesion and NSTEMI-A was angiographically diagnosed when at least 1 feature suggestive of a culprit lesion was present.

### Statistical Analysis

Continuous variables are expressed as means and standard deviations or medians and inter-quartile ranges, as appropriate. Categorical variables are presented as frequencies with percentages. The correlation between individual biomarkers was assessed using scatterplot analysis and the Pearson correlation coefficient was calculated.

The four biomarkers were combined to form a PDI using optimization of the area under the receiver operating characteristic curve (ROC AUC). The coefficients were computed using the formula of Su and Liu [26]. These weights would produce a PDI that has the maximum AUC among all other linear combinations of biomarkers assuming normal distribution of each biomarker. For ease of presentation, the weights were rescaled by the coefficient of the first biomarker in each PDI so that the weight equaled 1.0 for the first biomarker in each PDI. The values of MRP8/14 and MPO have been divided by 100 before forming the PDI.

ROC curves were constructed for each biomarker and PDI. A cut-point that optimizes specificity for the diagnosis of NSTEMI-A and differentiation of groups, conditional on achieving a minimum sensitivity of 80%, was determined. The sensitivity, specificity, NPV and positive predictive value (PPV) of the biomarkers, PDI, and angiographic criteria for establishing the diagnosis of type 1 MI were calculated using the clinical diagnosis as the gold standard and the aforementioned cut-point if applicable. Comparison of the diagnostic statistics between biomarkers, PDI and angiographic criteria was conducted using bootstrap analysis. A p value<0.05 was considered as statistically significant.

## Results

### Patient Characteristics

Baseline characteristics of the study groups are summarized in Table 1 and were comparable with the exception of a higher smoking rate in the NSTEMI-A group. Baseline therapy differed between the study groups, but was not different among the NSTEMI-A and NSTEMI-L groups. All NSTEMI-A patients had chest pain and a positive troponin. Nine patients (69.2%) had ECG changes meeting study criteria.

**Table 1:** Patient characteristics.

Variable	SNOCAD (n = 21)	SOCAD (n = 42)	NSTEMI-A (n = 15)	NSTEMI-L (n = 5)	P1	P2	P3
Age, years (Mean(SD))	64.9 (12.5)	66.3 (10.6)	63.5 (9.9)	65.6 (6.3)	0.828	0.364	0.662
Males, n (%)	10 (47.6)	33 (78.6)	13 (86.7)	3 (60.0)	0.031	0.495	0.249
Risk factors, n(%)							
HTN	14 (66.7)	31 (73.8)	11 (73.3)	4 (80.0)	0.939	0.971	1.000
DM	3 (14.3)	13 (31.0)	4 (26.7)	2 (40.0)	0.456	0.755	0.613
Dyslipidemia	13 (61.9)	30 (71.4)	8 (53.3)	4 (80.0)	0.527	0.202	0.603
Smoking Active	2 (9.5)	5 (11.9)	7 (46.7)	1 (20.0)	0.021	0.005	0.603
Former Smoker	8 (38.1)	10 (23.8)	1 (6.7)	3 (60.0)	0.051	0.149	0.032
Family history CAD	2 (9.5)	12 (28.6)	4 (26.7)	1 (20.0)	0.351	0.888	1.000
History, n(%)							
CABG	0 (0.0)	5 (11.9)	1 (6.7)	2 (40.0)	0.055	1.000	0.140
PVD	0 (0.0)	3 (7.1)	1 (6.7)	0 (0.0)	0.623	1.000	1.000
Investigations (Mean(SD)) EF (%)	58.7 (9.7)	55.3 (11.0)	53.9 (10.4)	51.6 (18.8)	0.230	0.460	0.825
Creatinine (μmol/L)	81.0 (18.0)	88.7 (25.8)	89.7 (30.3)	84.2 (20.2)	0.750	0.863	0.793
Therapy, n (%)							
Aspirin	15 (71.4)	36 (85.7)	15 (100.0)	5 (100.0)	0.097	0.325	-
Clopidogrel	5 (23.8)	13 (31.0)	13 (86.7)	5 (100.0)	0.000	<0.001	1.000
Beta-blockers	9 (42.9)	30 (71.4)	12 (80.0)	5 (100.0)	0.026	0.518	0.539
ACEI/ARB	8 (38.1)	21 (50.0)	14 (93.3)	5 (100.0)	0.001	0.003	1.000
Statin	9 (42.9)	29 (69.0)	13 (86.7)	4 (80.0)	0.037	0.183	1.000
Heparin	2 (9.5)	7 (16.7)	13 (86.7)	4 (80.0)	<0.001	<0.001	1.000

P-value is based on Kruskal–Wallis test, Fisher’s exact test or Chi-square test as appropriate.

P1-value is for the comparison across the four groups.

P2-value is for the comparison of SOCAD vs. NSTEMI-A.

P3-value is for the comparison of NSTEMI-A vs. NSTEMI-L.

### Descriptive summary of biomarkers

The data on troponin concentrations and time intervals for chest pain and biomarker measurement are shown in Table 2. The biomarker concentrations of the study groups are shown in Table 3. All biomarkers were significantly higher in the NSTEMI-A group with the exception of MRP8/14 (P = 0.179).

**Table 2:** Troponin concentrations and time intervals in NSTEMI-A patients.

	Median (IQR)	Mean (SD)	Range
**First troponin I concentration (μg/l)**	0.67 (0.16, 5.82)	11.53 (27.46)	(0.05, 100.00)
**Repeat troponin I concentration (μg/l)**	4.29 (1.14, 13.19)	14.83 (27.17)	(0.10, 100.00)
% change in troponin concentration (increase or decrease)*	126.63 (20.82, 612.50)	895.67 (2241.87)	(0.00, 8795.83)
**Chest pain to troponin duration (hrs:min)**	6:34 (3:55, 11:11)	11:38 (13:46)	(0:30, 48:00)
**Chest pain to biomarkers duration (hrs:min)**	21:28 (18:10, 27:10)	25:28 (12:23)	(13:37, 59:27)
**Troponin to biomarkers duration (hrs:min)**	14:19 (8:00, 19:25)	13:50 (6:09)	(2:32, 23:30)

* Percentage change between the first troponin and the repeat troponin.

**Table 3:** Single biomarker concentrations in the four study groups.

Biomarker	SNOCAD (n = 21)	SOCAD (n = 42)	NSTEMI-A (n = 15)	NSTEMI-L (n = 5)	P1	P2
**cTnI (μg/l)**^a^	0.02 (0.01)	0.02 (0.02)	12.59 (21.78)	1.21 (2.29)	0.137	<0.001
**PAPPA (μIU/mL)**	2.85 (3.38)	1.83 (0.88)	4.48 (3.25)	2.88 (2.40)	0.658	0.002
**MRP8/14 (μg/mL)**^b^	522.02 (434.68)	412.70 (311.02)	778.25 (823.33)	355.85 (340.74)	0.583	0.179
**Hs IL6 (pg/mL)**	2.82 (2.06)	2.90 (2.38)	6.57 (4.86)	2.78 (0.74)	0.686	<0.001
**MPO (pmol/L)**	753.96 (740.16)	635.05 (497.27)	1215.45 (630.64)	573.46 (181.16)	0.652	<0.001

P-value is based on Kruskal–Wallis test.

P1-value is for the comparison between SNOCAD, SOCAD and NSTEMI-L.

P2-value is for the comparison of NSTEMI-A vs. the other three groups combined.

^a^Simultaneous troponin value. Troponin values at the lower detection limit were considered to have a value 0.02 for the calculation of summary statistics.

^b^Data for MRP8/14 was missing for one NSTEMI-A subject.

Correlation between biomarkers was studied using scatterplot analysis, and Spearman’s correlation coefficient (Table 4) was calculated. A moderate correlation was present between MPO and PAPP-A, and between MPO and MRP8/14. Other biomarkers correlated mildly or poorly. Results on linear combinations of biomarkers in order to improve the diagnostic accuracy are shown in Table 5 and are significantly higher in the NSTEMI-A group (P<0.001).

**Table 4:** Spearman correlation between biomarkers.

	PAPPA	MRP8/14	hs IL6	MPO
**PAPPA**	1.00			
**MRP8/14**	0.25	1.00		
**hs IL6**	0.23	0.16	1.00	
**MPO**	0.41	0.40	0.30	1.00

**Table 5:** Linear combination of biomarkers–weights obtained by optimization of AUC.

Biomarker	SNOCAD (n = 21)	SOCAD (n = 42)	NSTEMI-A (n = 15)	NSTEMI-L (n = 5)	P1	P2
**PAPPA + 0.203 MRP8/14**	3.92 (3.42)	2.67 (1.23)	6.37 (3.91)	3.61 (2.74)	0.523	<0.001
**PAPPA + 1.196 Hs IL6**	6.23 (4.22)	5.29 (3.12)	12.33 (8.42)	6.20 (2.14)	0.352	<0.001
**PAPPA + 0.495 MPO**	6.59 (4.56)	4.97 (2.95)	10.50 (5.47)	5.72 (2.87)	0.244	<0.001
**MRP8/14 + 4.139 Hs IL6**	16.90 (10.45)	16.11 (10.75)	36.22 (23.08)	15.05 (1.92)	0.718	<0.001
**MRP8/14 + 12.765 MPO**	101.47 (96.14)	85.19 (63.90)	167.57 (87.23)	76.76 (25.57)	0.727	<0.001
**Hs IL 6 + 0.5436 MPO**	6.92 (4.23)	6.35 (3.79)	13.18 (6.70)	5.89 (0.44)	0.779	<0.001
**PAPPA + 0.359 MRP8/14 + 1.312 Hs IL6**	8.43 (4.76)	7.11 (3.78)	16.60 (9.90)	7.80 (2.51)	0.390	<0.001
**PAPPA + 0.0204 MPR8/14 + 0.486 MPO**	6.62 (4.53)	5.00 (2.92)	11.03 (5.37)	5.74 (2.89)	0.265	<0.001
**PAPPA + 2.006 Hs IL6 + 1.200 MPO**	17.56 (9.31)	15.25 (8.43)	32.23 (16.52)	15.33 (2.74)	0.509	<0.001
**MPR8/14 + 2.248 Hs IL6 + 3.836 MPO**	40.48 (30.07)	35.00 (21.01)	71.24 (34.96)	31.80 (8.03)	0.827	<0.001
**PAPPA + 1.441 MRP8/14 + 3.524 Hs IL6 + 5.942 MPO**	65.12 (45.90)	55.71 (32.91)	114.59 (55.46)	51.87 (13.23)	0.650	<0.001

Results represent means (standard deviations).

P-value is based on Kruskal–Wallis test.

P1-value is for the comparison between SNOCAD, SOCAD and NSTEMI-L.

P2-value is for the comparison of NSTEMI-A vs. the rest of the three groups combined.

The values of MRP8/14 and MPO have been divided by 100 before combining.

Data for MRP8/14 was missing for one NSTEMI-A subject.

When individual markers were compared, MPO was the marker with the highest specificity (84%) for the identification of patients with Non-STE-type 1 MI when measured within 24 hours from the first detectable troponin (AUC 0.83, accuracy 83%) (Table 6) (P<0.05). The lowest specificity was observed with MRP8/14. When biomarker combinations were analysed, the highest specificity was obtained by the combination of PAPP-A, MPR8/14 and MPO. This resulted in a specificity of 87%, sensitivity of 79%, accuracy of 86% and an AUC of 0.84, which were higher compared to those obtained with MPO but did not reach statistical significance (P>0.05) for all comparisons. Use of all markers increased the AUC to 0.88, but did not result in any improvement in specificity (86%).

**Table 6:** Diagnostic accuracy of biomarkers, coronary angiography and combinations.

Single biomarkers	AUC	Cut-point	Specificity	Sensitivity^b^	NPV	PPV	Accuracy
**PAPPA**	0.77 (0.60, 0.93)	2.078	0.67 (0.55, 0.78)^c^	0.80 (0.57, 1.00)	0.93 (0.85, 1.00)	0.36 (0.21, 0.54)	0.69 (0.59, 0.79)
**MRP8/14**	0.61 (0.43, 0.78)	280.674	0.40 (0.27, 0.52)^c^	0.79 (0.55, 1.00)	0.89 (0.76, 1.00)	0.22 (0.12, 0.35)	0.47 (0.35, 0.58)
**Hs IL6**	0.80 (0.69, 0.92)	3.064	0.68 (0.56, 0.80)^c^	0.80 (0.57, 1.00)	0.93 (0.86, 1.00)	0.38 (0.21, 0.55)	0.71 (0.61, 0.81)
**MPO**	0.83 (0.71, 0.95)	885.501	0.84 (0.75, 0.93)^c^	0.80 (0.58, 1.00)	0.95 (0.88, 1.00)	0.55 (0.33, 0.75)	0.83 (0.75, 0.91)
**PDI (by optimization of AUC** ^a^**)**							
PAPPA + 0.203 MRP8/14	0.82 (0.68, 0.95)	4.042	0.81 (0.71, 0.91)	0.79 (0.57, 1.00)	0.94 (0.88, 1.00)	0.48 (0.27, 0.69)	0.81 (0.72, 0.89)
PAPPA + 1.196 Hs IL6	0.83 (0.71, 0.94)	7.161	0.78 (0.68, 0.88)	0.80 (0.57, 1.00)	0.94 (0.87, 1.00)	0.46 (0.27, 0.67)	0.78 (0.69, 0.87)
PAPPA + 0.495 MPO	0.81 (0.66, 0.95)	7.107	0.84 (0.75, 0.92)	0.80 (0.58, 1.00)	0.95 (0.88, 1.00)	0.55 (0.33, 0.75)	0.83 (0.75, 0.91)
MRP8/14 + 4.139 Hs IL6	0.83 (0.73, 0.94)	18.674	0.73 (0.62, 0.84)	0.79 (0.56, 1.00)	0.94 (0.87, 1.00)	0.39 (0.22, 0.58)	0.74 (0.64, 0.84)
MRP8/14 + 12.765 MPO	0.84 (0.72, 0.96)	120.011	0.84 (0.75, 0.93)	0.86 (0.67, 1.00)	0.96 (0.91, 1.00)	0.55 (0.33, 0.75)	0.84 (0.76, 0.92)
Hs IL 6 + 0.5436 MPO	0.86 (0.78, 0.95)	8.343	0.76 (0.65, 0.87)	0.80 (0.57, 1.00)	0.94 (0.87, 1.00)	0.44 (0.26, 0.64)	0.77 (0.67, 0.86)
PAPPA + 0.359 MRP8/14 + 1.312 Hs IL6	0.87 (0.80, 0.95)	9.848	0.79 (0.69, 0.89)	0.79 (0.56, 1.00)	0.94 (0.88, 1.00)	0.46 (0.26, 0.65)	0.79 (0.70, 0.88)
PAPPA + 0.0204 MPR8/14 + 0.486 MPO	0.84 (0.70, 0.97)	8.156	0.87 (0.78, 0.95)^d^	0.79 (0.57, 1.00)^d^	0.95 (0.89, 1.00)^d^	0.58 (0.36, 0.81)^d^	0.86 (0.78, 0.93)^d^
PAPPA + 2.006 Hs IL6 + 1.200 MPO	0.86 (0.76, 0.95)	21.666	0.78 (0.67, 0.88)	0.87 (0.67, 1.00)	0.96 (0.90, 1.00)	0.48 (0.30, 0.68)	0.79 (0.70, 0.88)
MPR8/14 + 2.248 Hs IL6 + 3.836 MPO	0.87 (0.78, 0.96)	49.594	0.86 (0.76, 0.94)	0.79 (0.54, 1.00)	0.95 (0.88, 1.00)	0.55 (0.33, 0.77)	0.84 (0.76, 0.92)
PAPPA + 1.441 MRP8/14 + 3.524 Hs IL6 + 5.942 MPO	0.88 (0.78, 0.97)	79.894	0.86 (0.76, 0.94)	0.79 (0.54, 1.00)	0.95 (0.88, 1.00)	0.55 (0.33, 0.77)	0.84 (0.76, 0.92)
**Angiographic criteria**							
C1—Ulceration	-	-	0.95 (0.89, 1.00)	0.07 (0.00, 0.25)	0.82 (0.73, 0.91)	0.25 (0.00, 1.00)	0.79 (0.70, 0.88)
C2—Lumen irregularity	-	-	0.86 (0.77, 0.94)	0.36 (0.10, 0.64)	0.86 (0.77, 0.94)	0.36 (0.09, 0.64)	0.77 (0.67, 0.86)
C3—Intimal flap	-	-	0.97 (0.92, 1.00)	0.07 (0.00, 0.23)	0.82 (0.73, 0.91)	0.33 (0.00, 1.00)	0.81 (0.72, 0.89)
C4—Impairment in wall motion	-	-	0.92 (0.85, 0.98)	0.29 (0.07, 0.55)	0.85 (0.76, 0.93)	0.44 (0.11, 0.80)	0.81 (0.71, 0.89)
C5—Flow impairment	-	-	0.81 (0.70, 0.90)	0.36 (0.10, 0.64)	0.85 (0.75, 0.93)	0.29 (0.08, 0.53)	0.73 (0.62, 0.82)
C6 –Occlusive thrombus	-	-	0.84 (0.75, 0.92)	0.29 (0.06, 0.54)	0.84 (0.75, 0.93)	0.29 (0.07, 0.54)	0.74 (0.64, 0.83)
C7—Sub-totally occlusive thrombus	-	-	0.83 (0.73, 0.92)	0.57 (0.31, 0.82)	0.90 (0.81, 0.97)	0.42 (0.20, 0.65)	0.78 (0.68, 0.87)
C8—Any thrombus	-	-	0.68 (0.56, 0.79)	0.71 (0.45, 0.93)	0.91 (0.83, 0.98)	0.33 (0.16, 0.50)	0.69 (0.58, 0.79)
C9—Presence of any feature	-	-	0.59 (0.46, 0.70)	0.86 (0.64, 1.00)	0.95 (0.86, 1.00)	0.32 (0.17, 0.47)	0.64 (0.52, 0.74)
C10—Presence of ≥2 features	-	-	0.73 (0.61, 0.83)	0.43 (0.17, 0.70)	0.85 (0.75, 0.94)	0.26 (0.09, 0.44)	0.68 (0.56, 0.77)
C11—Presence of ≥3 features	-	-	0.95 (0.89, 1.00)	0.29 (0.07, 0.55)	0.86 (0.77, 0.93)	0.57 (0.14, 1.00)	0.83 (0.74, 0.91)

^a^For PDI, MRP8/14 and MPO have been divided by 100 before combining.

Diagnostic statistics was based on the clinical diagnosis of NSTEMI-A (n = 15) vs stable patients (n = 63). Data for MRP8/14 was missing for one NSTEMI-A subject. Data for angiographic diagnosis was missing for one other NSTEMI subject. Values in brackets are 95% CI based on bootstrapping. Subjects with biomarker greater than the cut point are classified as having NSTEMI for the calculation of diagnostic statistics. 2. Angiographic criteria was entered as a 0/1 variable in the PDI.

^b^ Sensitivity might not be exactly 0.8 for the cut-point due to finite sample size. If the same specificity was achieved by multiple cut-points, the one with the highest sensitivity was chosen.

^c^P<0.05 for comparison with MPO.

^d^P>0.05 for comparison with MPO alone.

### Angiographic Features and Diagnosis

Table 7 shows the angiographic results of the study subjects. The NSTEMI-A and NSTEMI-L groups were comparable in all angiographic parameters. Among the SOCAD and NSTEMI-A groups, no significant differences were observed in the average number of diseased vessels, percent DS, stenosis of worst lesion, and coronary atherosclerotic burden as evaluated by the COURAGE Jeopardy score. There were no significant differences in the predefined angiographic criteria for plaque instability between the SOCAD and NSTEMI-A groups except for the presence of sub-totally occlusive thrombus (P = 0.034). When analyzed by the presence of any criteria of instability, only the presence of ≥3 criteria discriminated the 2 groups (P = 0.036).

**Table 7:** Angiographic features of the study population.

Variable	SNOCAD (n = 21)	SOCAD (n = 42)	NSTEMI-A (n = 15)	NSTEMI-L (n = 5)	P1	P2	P3
**Vessel disease (>50%), mean (SD)**	0.00 (0.00)	1.95 (0.91)	1.93 (0.88)	2.20 (0.84)	<0.001	0.969	0.576
**% Diameter stenosis**^a^ **mean (SD)**	31.4 (8.0)	64.8 (14.5)	61.8 (13.7)	59.9 (18.3)	<0.001	0.528	0.631
**% Stenosis of worst lesion mean (SD)**	28.4 (15.5)	80.4 (18.9)	80.3 (17.9)	71.4 (21.1)	<0.001	0.888	0.376
**Jeopardy score, mean (SD)**	0.0 (0.0)	8.3 (5.9)	9.4 (4.9)	9.8 (7.0)	<0.001	0.462	0.694
**Ulceration, n (%)**^b^	0 (0.0)	3 (7.1)	1 (7.1)	0 (0.0)	0.614	1.000	1.000
**Lumen irregularity, n (%)**^b^	0 (0.0)	9 (21.4)	5 (35.7)	4 (80.0)	<0.001	0.285	0.141
**Intimal flap, n (%)**^b^	0 (0.0)	2 (4.8)	1 (7.1)	0 (0.0)	0.656	1.000	1.000
**Impairment in wall motion, n (%)**^b^	0 (0.0)	5 (11.9)	4 (28.6)	1 (20.0)	0.044	0.141	1.000
**Flow impairment, n (%)**^b^	0 (0.0)	12 (28.6)	5 (35.7)	2 (40.0)	0.007	0.615	1.000
**Thrombus formation, n (%)**^b^							
**Occlusive**	0 (0.0)	10 (23.8)	4 (28.6)	1 (20.0)	0.033	0.722	1.000
**Sub-totally occlusive**	0 (0.0)	11 (26.2)	8 (57.1)	4 (80.0)	<0.001	0.034	0.603
**Any thrombus**	0 (0.0)	20 (47.6)	10 (71.4)	4 (80.0)	<0.001	0.122	1.000
**Presence of any feature suggestive of a culprit lesion, n (%)**^b^	0 (0.0)	26 (61.9)	12 (85.7)	5 (100.0)	<0.001	0.099	1.000
**Presence of ≥2 features in a single vessel, n (%)**^b^	0 (0.0)	17 (40.5)	6 (42.9)	3 (60.0)	<0.001	0.875	0.628
**Presence of ≥3 features in a single vessel, n (%)**^b^	0 (0.0)	3 (7.1)	4 (28.6)	1 (20.0)	0.026	0.036	1.000

P-value is based on Kruskal–Wallis test, Fisher’s exact test or Chi-square test as appropriate.

P1-value is for the comparison across the four groups.

P2-value is for the comparison of SOCAD vs. NSTEMI-A.

P3-value is for the comparison of NSTEMI-A vs. NSTEMI-L.

^a^Exclude segments with 0%. Values from multiple segments were first averaged within each subject and the resulting averages were used to produce the summary statistics for each group.

^b^Data missing for one NSTEMI-A patient.

**Fig 1:**
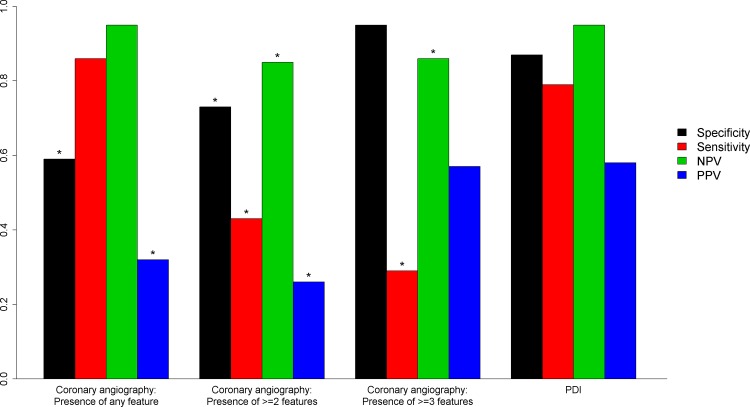
Diagnostic performance of the PDI and coronary angiography in confirming the diagnosis of NSTE-type 1 MI. * = P<0.05 for comparison with PDI.

The angiographic criteria overall had low sensitivity for NSTEMI-A diagnosis despite high specificity. The NPV, PPV and accuracy were comparatively lower (Table 6). The PDI had higher NPV compared to the presence of two or three angiographic criteria in any single vessel. (Table 6 and Fig 1).

## Discussion

Our findings suggest that an index calculated from multiple markers of plaque destabilization and disruption measured within 24 hours of cTn positivity has the potential to identify subjects with Non-STE-Type 1 MI. Using a cut-point based on fixed sensitivity a PDI computed from PAPPA, MPR8/14 and MPO had the highest specificity and accuracy compared to individual markers, however, this did not reach statistical significance in comparison with MPO (P = 0.302 and P = 0.332 respectively). Our results also suggest that compared with prespecified standard coronary angiography criteria, the PDI showed superior specificity for identifying subjects with Non-STE-Type 1 MI.

**Fig 2:**
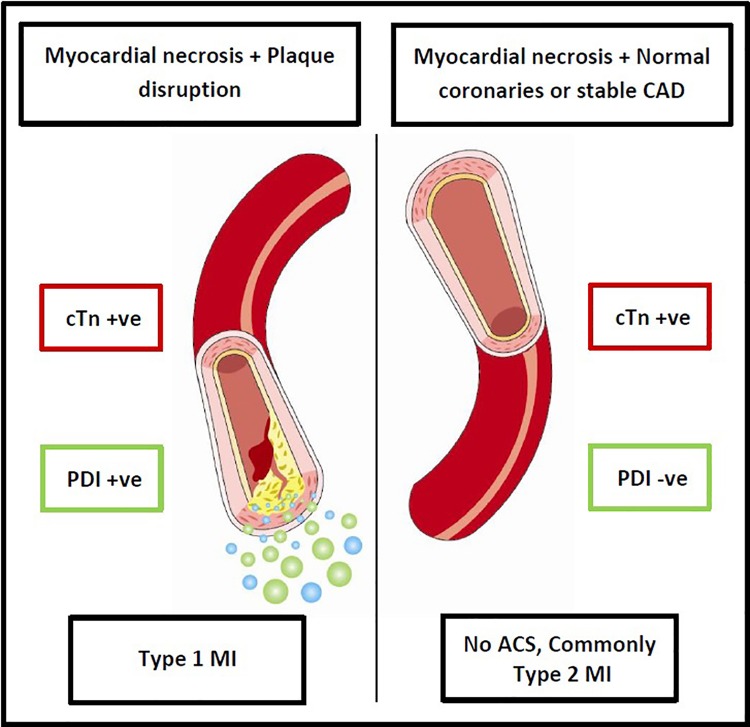
Hypothesized role of the PDI in distinguishing type 1 and type 2 MI. cTn = troponin, PDI = plaque disruption index, MI = myocardial infarction.

Unique to our study is (1) the calculation of an index from multiple markers and (2) measurement of the biomarkers’ systemic concentrations within a predetermined time limit specified from the first clinically detectable troponin rather than from symptom onset. We have elected to use this time frame in consideration of the potential future investigation of the index in discriminating NSTE-Type 1 MI from non-ACS related troponin elevations (most commonly, Type 2 MI) in patients with cTn elevation of uncertain etiology. A negative PDI in patients with troponin elevation but at low risk of ACS may effectively rule out ACS in a similar manner to the current application of the d-dimer test in ruling out pulmonary embolism (Fig 2). This study provided important data regarding (1) the “downstream” diagnostic value of the biomarkers in ACS, (2) the specification of a time frame for the measurement of biomarker concentrations, and (3) the establishment of diagnostic cut-off values for the biomarkers and PDI within that time frame. This information is essential when considering to evaluate the biomarkers’ diagnostic value in subjects with troponin elevation of uncertain etiology.

Our results on the sensitivity and specificity of individual biomarkers are comparable to those described previously with MPO [20] and within those previously reported for PAPP-A [19; 27]. The diagnostic role of MRP8/14 in ACS has been reported once previously [18] and our results are lower possibly due to the later timing of measurement. The role of hsIL-6 in establishing an ACS diagnosis has also not been previously reported, although elevations in ACS are well described [22; 23; 28].

The lack of superior specificity of the PDI compared to MPO in our study does not negate the PDI value. The small patient number may have contributed to the lack of statistical significance when the PDI and individual biomarkers were compared. It is also worthwhile to investigate the role of other plaque disruption markers in a PDI which can be used to substitute the less specific markers in our study. The superiority of MPO may be attributed to its role in the pathogenesis of plaque disruption. Although increases in plaque MPO concentrations may promote either erosion or rupture, the density of MPO-positive cells is significantly higher in thrombi overlying superficial erosions [15], a common cause of NSTEMI presentation [29], and the systemic levels of MPO parallel this difference [15]. PAPP-A is abundantly expressed in both ruptured and eroded plaques [19], while the concentrations of IL-6 and MRP8/14 have not been compared in the different types of culprit lesions [2].

Further investigations into the role of biomarkers in the pathogenesis of plaque disruption and their local and systemic concentrations are warranted. Inclusion of markers which are particularly elevated in patients with eroded plaques in a PDI may be worthwhile. Development of new and specific plaque disruption markers is also important. Pending the identification of the ideal plaque disruption marker; an index may be the best available alternative being non-invasive and feasible.

Prior studies involving these markers in ACS have shown that circulating levels vary with culprit lesion morphology [15], severity of clinical presentation (highest in STEMI) [18; 19; 21] and time from symptom onset [21], with decreasing sensitivity and specificity as the time increases [20]; the latter may explain the low concentrations observed in the NSTEMI-L group. Additionally, commercially available assays vary in their cut-off values and diagnostic sensitivities and specificities [30]. Naturally, since the release of these markers is not exclusive for ACS, differences in sensitivities and specificities are expected to vary according to the studied populations. The PDI model should therefore be validated in a large non-selected cohort. We hypothesize that the PDI, being derived from multiple markers with different tissue specificities, may retain its diagnostic accuracy when applied to non-selected patient populations. This however remains to be tested.

Complex angiographic lesions correlate with disrupted plaques pathologically and presentation with ACS clinically [25]. Our results show that angiographic criteria overall had very low sensitivity for NSTEMI diagnosis despite high specificity. Coronary angiography was most useful in ruling out NSTEMI in the cases where there was <50% coronary stenosis. This however may not be very useful clinically. Blich et al found that 77% of the patients with non-ACS-related troponin elevations have significant flow-limiting CAD, with over half having three-vessel disease [6]. Because CAD is common, the routine referral for coronary angiography to exclude non-ACS–related troponin elevation, where clinical diagnosis is not clear, may not be the best clinical option. While the sensitivity of intravascular coronary imaging may be higher, it remains suboptimal for the detection of culprit lesions in ACS [31], and may not be a practical alternative in daily practice.

Our study is limited by the small number of patients. Additionally, due to the study design, it was not possible to exclude patients receiving heparin, which was administered to all NSTEMI patients at the time of enrollment. Concomitant heparin administration is suggested to increase biomarker levels, but the exact effect of is not fully delineated. Although MPO plasma levels increase in patients receiving heparin, it retains its diagnostic value [21]. Conflicting effects are reported with PAPP-A [32; 33]. In our study, comparison of NSTEMI-A with NSTEMI-L patients (all receiving heparin) revealed higher levels in the acute group, with comparable biomarker levels in the NSTEMI-L and stable CAD patients. We think that the time from ACS onset, probably had a greater influence on biomarker levels than concomitant heparin administration, with decreasing levels as the time from ACS onset increases. The patient numbers are too small, however to make conclusions in that regard.

## Conclusions

A PDI calculated from PAPP-A, MPO and MRP8/14 and measured within 24 hours of cTn positivity in patients with acute NSTE-type 1-MI has the potential to confirm this diagnosis but did not yield higher specificity nor negative predictive value compared to MPO. Additional evidence with the use of other markers, in a PDI and assessment in a sufficiently large non-selected cohort of patients is warranted to establish the diagnostic accuracy of the PDI. Development of a more specific PDI may be of value in confirming type 1 MI diagnosis and differentiating type 1 and type 2 MI. Further studies to investigate its utility are warranted.
